# Supplementary material for: Building programmable multicompartment artificial cells incorporating remotely activated protein channels using microfluidics and acoustic levitation
Source: Nat Commun. 2022 Jul 15;13:4125. doi: 10.1038/s41467-022-31898-w (PMC9287423; doi:10.1038/s41467-022-31898-w)
Supplement: Supplementary file 1 — Supplementary Information [file 41467_2022_31898_MOESM1_ESM.pdf]

Building programmable multicompartment artificial cells incorporating remotely activated protein channels using microfluidics and acoustic levitation

Jin Li[1]*, William D. Jamieson[2], Pantelitsa Dimitriou[1], Wen Xu[3], Paul Rohde[4], Boris Martinac[4,5], Matthew Baker[6], Bruce W. Drinkwater[7]*, Oliver K. Castell[2]*, David A. Barrow[1]*

[1]School of Engineering, Cardiff University, The Parade, Cardiff, CF24 3AA, U.K.
[2]School of Pharmacy and Pharmaceutical Sciences, Cardiff University, King Edward VII Ave, Cardiff, CF10 3NB, U.K.
[3]Cardiff Business School, Cardiff University, Aberconway Building, Colum Dr, Cardiff, CF10 3EU, U.K.
[4]Victor Chang Cardiac Research Institute, Lowy Packer Building, 405 Liverpool St, Darlinhurst, NSW 2010, Australia.
[5]School of Clinical Medicine, UNSW, Sydney, NSW, 2052, Australia.
[6]School of Biotechnology and Biomolecular Science, UNSW, Sydney, NSW, 2052, Australia.
[7]Department of Mechanical Engineering, University of Bristol, University Walk, Bristol, BS8 1TR, U.K.

Content of supplementary materials

Fig S1. Droplet laboratory experiment setup

Fig S2. Experimental and simulated droplet trajectories in the acoustic trap

Fig S3. The relationship between levitator input voltage and the levitated droplet shape

Fig S4. Droplet interface lipid bilayer formation between water compartments in a levitated, lipid containing oil droplet

Fig S5. Simulated and experimental details of 3D-printed, multi-layered, droplet forming fluidic junctions.

Fig S6. Complementary data of pneumatic operations for the spinning of levitated ACDC droplets (Fig 3B).

Fig S7. Shell photo-polymerisation of a microfluidically-formed, levitated oil/PEGDA multi-phase droplet.

Fig S8. Schematics of protein reconstitution in levitated ACDC droplets.

Fig S9. Relative fluorescent intensity of the Fluo-8 containing cores (white) with different concentrations of pre-loaded $CaCl_2$ in levitated ACDC droplets.

Fig S10. Evaporation effects on fluorescence intensity are minimal compared to Ca^{2+} transport over the course of 30 minutes.

Fig S11. Control experiment of MscL gating by asymmetric DOPC-DPhPC bilayers.

Fig S12. Simulated results of tension within the droplet network of levitated ACDC droplet.

Fig S13. Relative intensity of a control core (white dotted circle) containing fluo-8 but no MscL, in a levitated ACDC droplet.

SI Video 1. Levitation of ACDC droplet

SI Video 2. Contactless operation of ACDC droplet

SI Video 3. MscL protein channel activation

Supplementary Figures

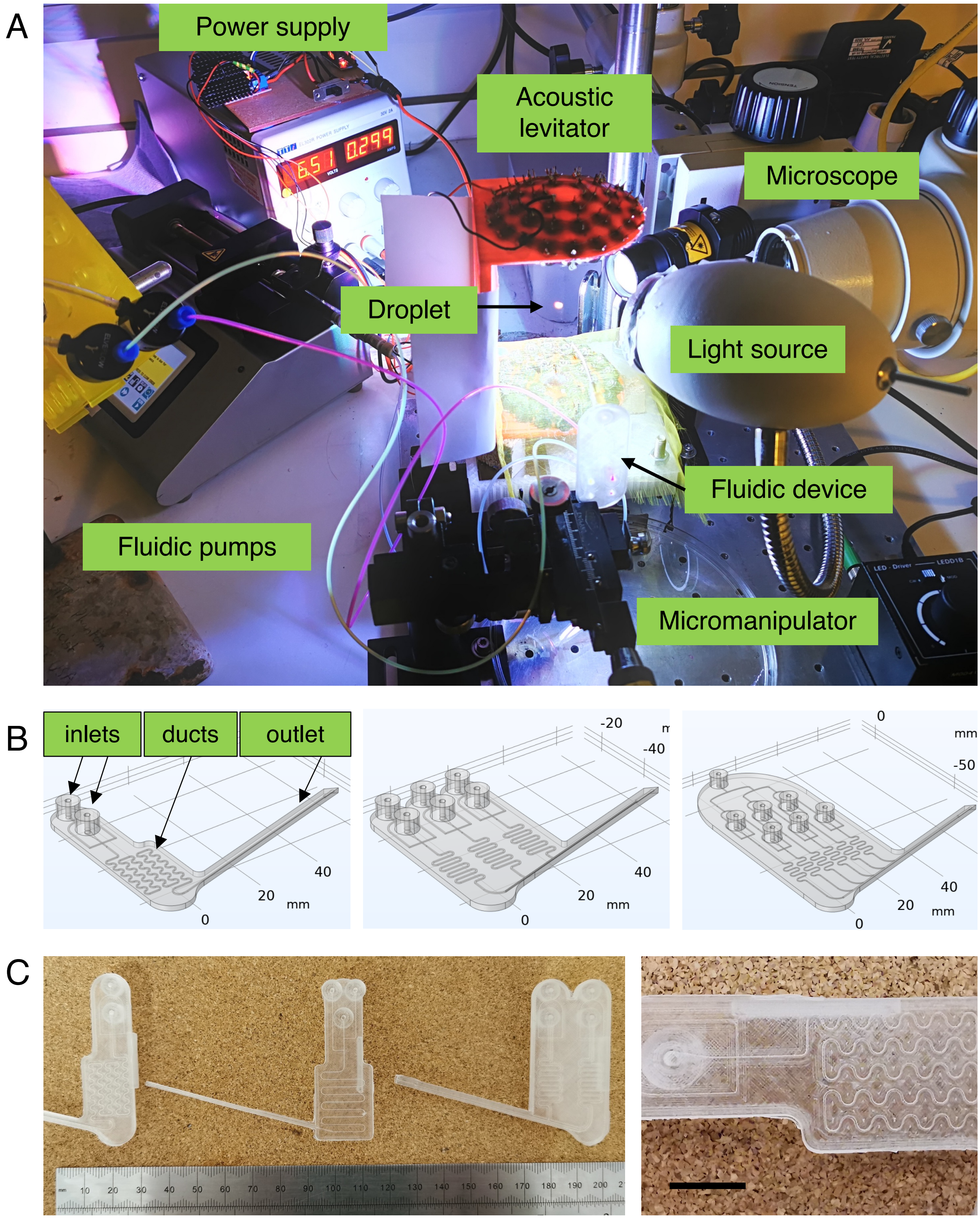

Fig S1. Droplet laboratory experiment setup. **A.** Setup overview - an ACDC droplet can be seen levitating in the acoustic trap. **B.** CAD Images of microfluidic device. From left to right, 1, 3, 6 cross-shaped droplet forming junctions in microfluidic circuits. **C.** Examples of 3D-printed COC microfluidic devices. Scale bar = 1 cm.

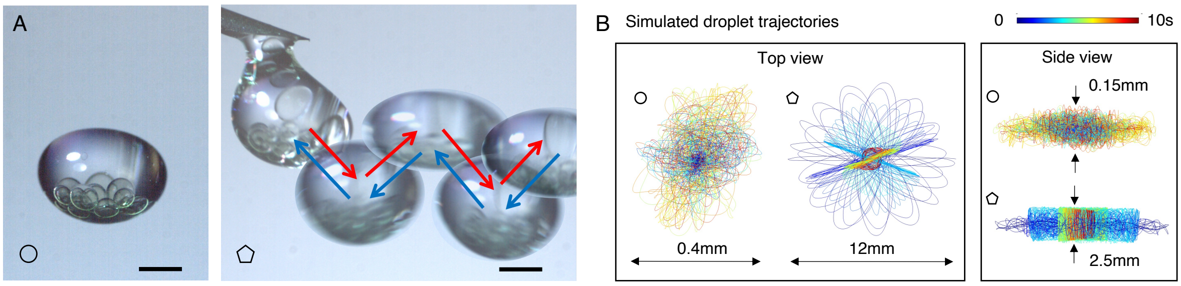

Fig S2. Experimental and simulated droplet trajectories in the acoustic trap, data complementary to Figure 1C. **A.** Left, an ACDC droplet stably levitated in air in the centre of an acoustic trapping node. Right, the trajectory (red followed by blue arrows) of a levitated ACDC droplet released from a position offset from the centre of the acoustic standing wave. Scale bars = 1 mm. **B.** Simulated trajectories of levitated droplets in comparison to that shown in Fig S2A. The trajectories marked with circles indicate that the droplet displays small oscillations both horizontally (< 200 µm) and vertically (<75 µm), even if levitated relatively stable in the acoustic standing wave. The trajectories marked with pentagons indicate the simulated trajectories when the droplet is released from an offset position as illustrated experimentally in Fig. S2A-right panel (pentagon labelled), where much larger oscillatory ranges are observed (6 mm).

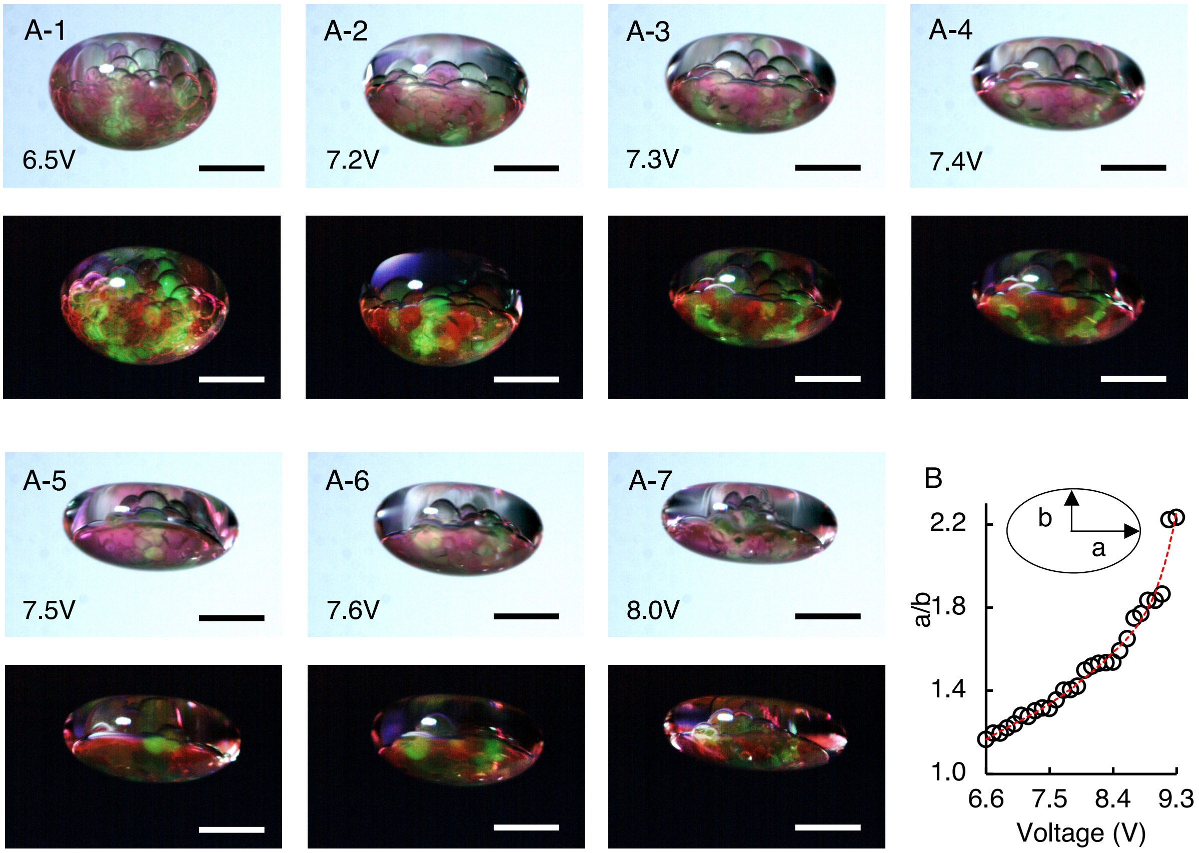

Fig S3. The relationship between levitator input voltage and the levitated droplet shape. A1-7. Example bright and dark images shows that the shape of a levitated ACDC droplet, which contained two types of cores (red and green), was gradually changed from ellipsoid to disk with progressive increases of input voltage and thus acoustic power to the levitator. Note that lipid bilayers remain stable throughout the shape manipulation with no coalescence of the cores. **B.** Measurement of the droplet aspect ratio (a/b) with increasing acoustic power (voltage to levitator) over the range 6.6. to 9.3 V. Representing quantitative analysis of the images of the type shown in A1-7. Scale bars = 1 mm.

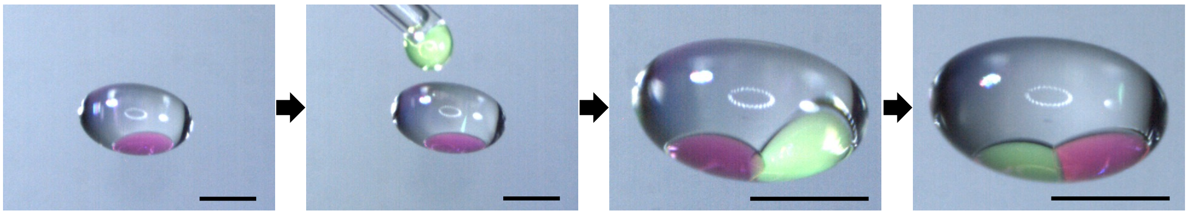

Fig S4. Droplet interface lipid bilayer formation between water compartments in a levitated, lipid containing oil droplet. From left to right, a 0.4 μL, calcein containing aqueous droplet (green) was manually added to a 6 μL levitated, hexadecane droplet containing 8 mg/mL DOPC, encapsulating another aqueous droplet with sulforhodamine B (pink). Lipids self-assembled at the water and oil interface. As shown in the most right image, droplet interface lipid bilayer was formed between green and pink water droplets, evidenced by their flat interface and the intact compartments without mixing. Scale bars = 1 mm.

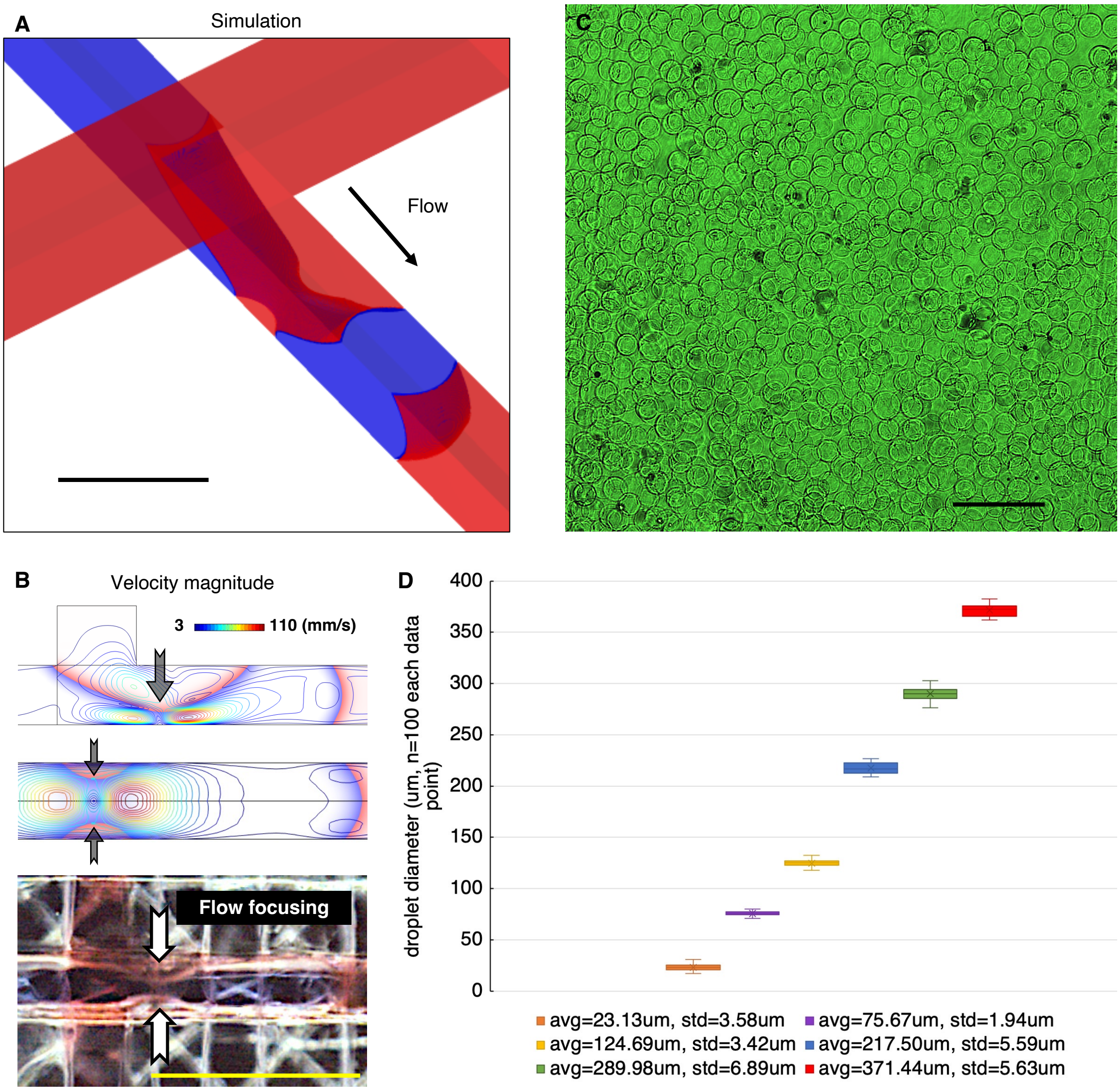

Fig S5. Simulated and experimental details of 3D-printed, multi-layered, droplet forming fluidic junctions. The multi-layered fluidic droplet forming junctions were designed to have the two input fluidic ducts stack on each other orthogonally, with a rectangular interface. This is to have sharp corners of the junction by shaping the interface with two straight printed lines, in comparison to the single layer junction that will naturally have roundish corners due to the movement resolution of the printhead. **A**. COMSOL simulation of water droplet (blue, 0.1 ml hr^{-1}) at the point of break up in a continuous oil phase flow (red, 0.8 ml hr^{-1}). Scale bar = 300 μm. **B.** Top, simulated velocity profiles during droplet breakup (side view and top view). The black arrows indicate the focusing flow. Bottom, corresponding image of water droplet (blue) break up in a mineral oil continuous phase flow (red) in a 3D-printed fluidic device. Scale bar = 0.5 mm. The visible cross-hatching effect is a consequence of the deposition lines of the polymer 3D printing process. **C.** A collection of alginate (average diameter = 124.69 μm) microgels formed by a multi-layered junction as illustrated in A and B. Scale bar = 1 mm. **D.** The uniformity of the droplet formed by the multi-layered junction, diameters ranging from 20 μm (approximate cell size) to 370 μm, which is controlled by the input flow rates ratio. Error bars indicate the standard error of mean of the droplet size.

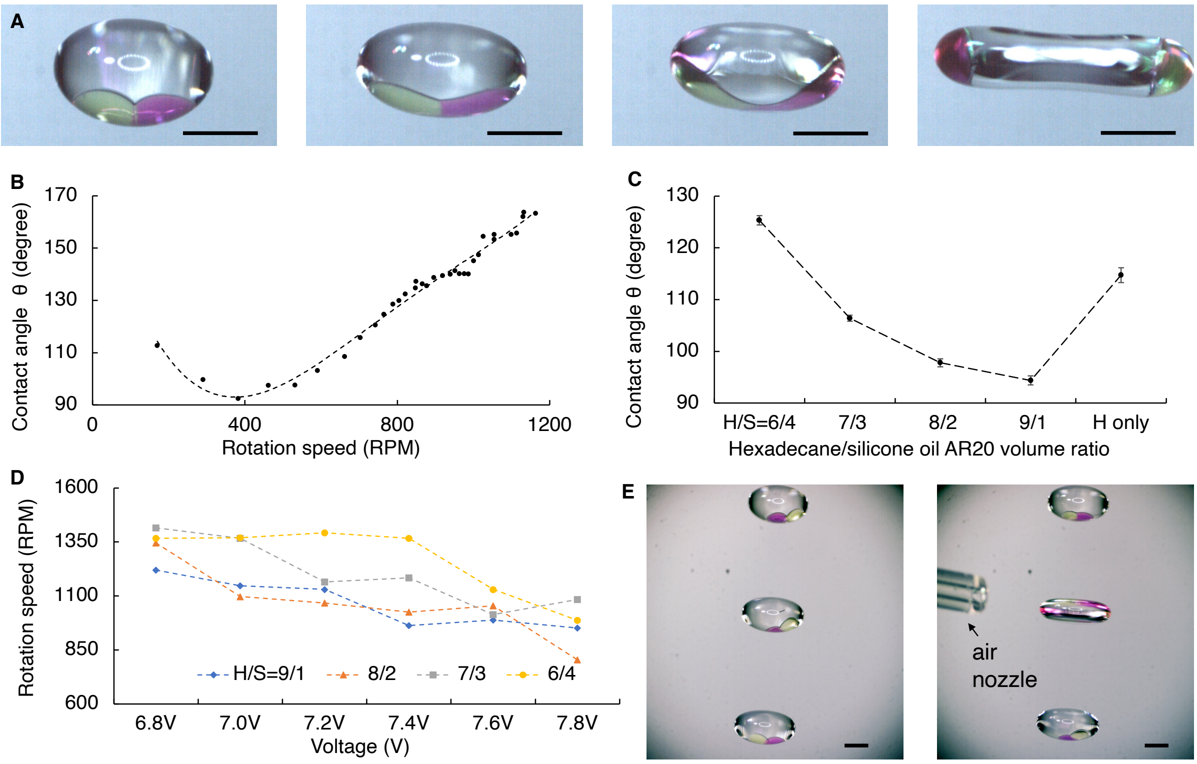

Fig S6. Complementary data of pneumatic operations for the spinning of levitated ACDC droplets (Fig 3B). Scale bars = 1 mm. **A.** From left to right, sequence of images showing aqueous cores (green and pink) connected by a lipid bilayer. Upon increasing spin speed due to increased air flow, the aqueous droplets move radially outwards in the less dense oil phase, reducing the bilayer area before eventually detaching. During the spinning action, the ACDC droplet shape progressively transforms from ellipsoid to a dumbbell shape, with the previously connected cores (green and pink) now spatially separated and residing at opposite ends of the dumbbell. **B.** The change of contact angle between the green and pink cores with increased spin rate of ACDC droplets. **C**. Contact angle of the red and green cores in different oil hexadecane (H) and silicone oil (S) mixtures. Error bars indicate the standard error of mean of the contact angle. **D**. The spin rate of ACDC droplets under different applied voltages that gives rise to internal green and pink droplet detachment. Shown for different hexadecane and silicone oil mixtures. **E**. On levitation of multiple ACDC droplets in sequential nodes of the levitator, the pneumatic operation can be applied to a specific ACDC droplet without significantly influencing those levitating in neighbouring nodes of the levitator's single axis acoustic standing wave field. Here a shape manipulation and droplet bilayer detachment is demonstrated on the middle ACDC droplet in a series of three.

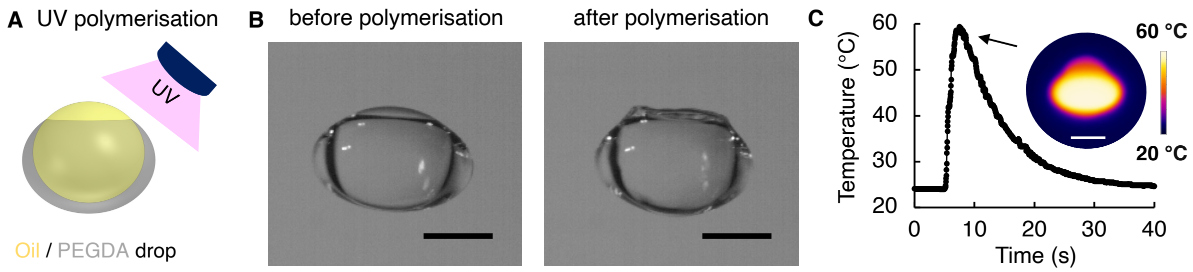

Fig S7. Shell photo-polymerisation of a microfluidically-formed, levitated oil/PEGDA multi-phase droplet. A. Schematic of the operation. **B.** images before and after the polymerisation. A bucket shaped solid shell was polymerised from the liquid PEGDA containing 1 wt% Irgacure 369 photoinitiator. **C.** The heat generated by the photopolymerisation and its dissipation during the reaction measured *in situ* using a thermal-imaging camera. Scale bars = 1 mm.

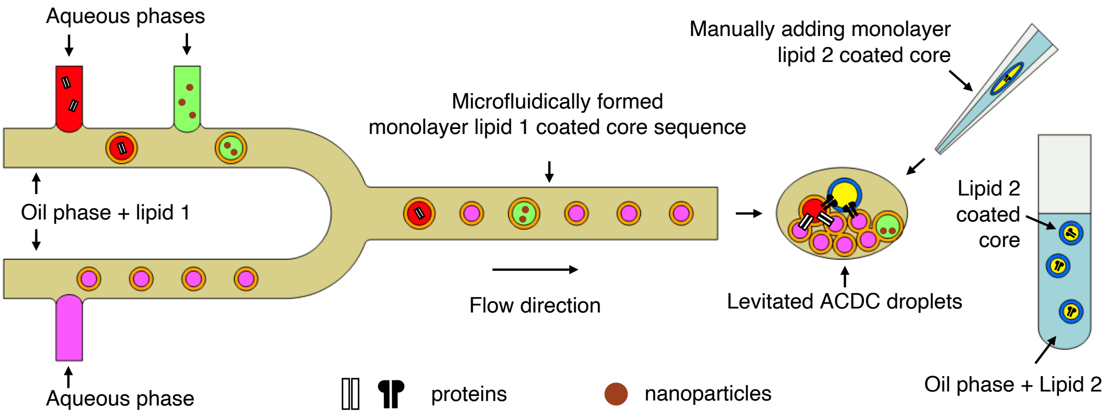

Fig S8. Schematic illustrating protein reconstitution in levitated ACDC droplets. Lipid coated water droplets (orange shell and blue shell spheres) were formed in oil phases containing dissolved lipid, either within a microfluidic device (left) or via manual preparation (right). Water droplets encapsulated either nanoparticles, proteins or chemical reagents. Lipid droplet interface bilayers were formed once the lipid-monolayer coated water droplets contacted each other, with the bilayer leaflet composition of each determined by the incubating lipid in oil solution, thus enabling the preparation of asymmetric bilayers when required. Single droplet addition allows for ultra-low volume reagent usage, e.g. for high-value reagents. The membrane proteins provided in the aqueous droplet spontaneously reconstitute to the formed bilayers [1].

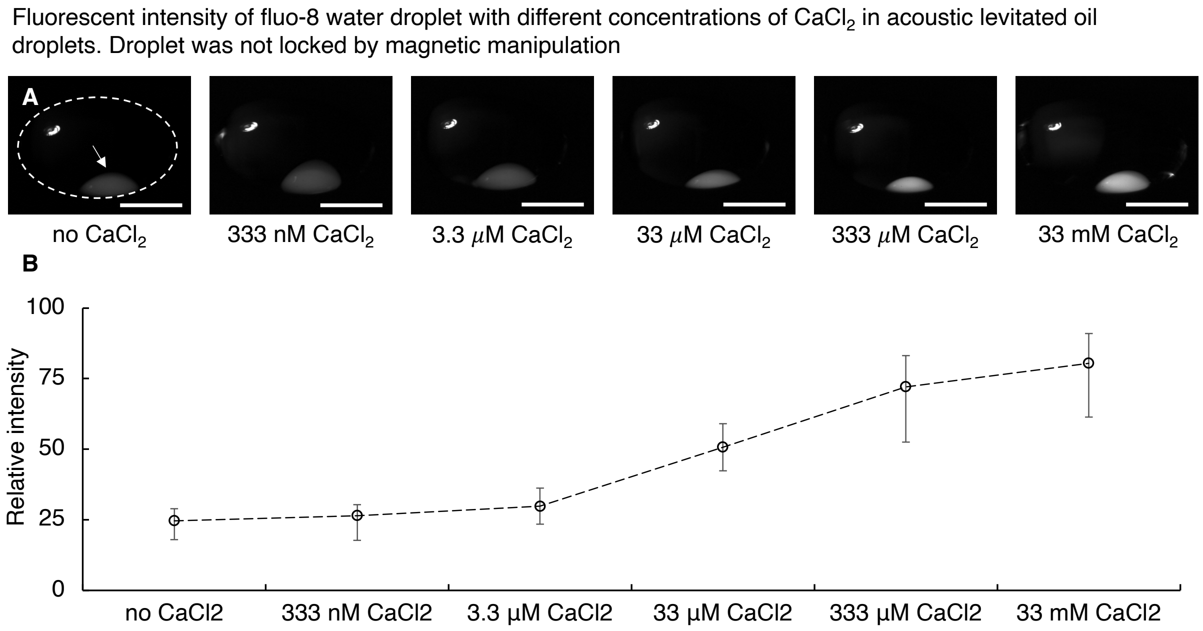

Fig S9. Relative fluorescent intensity of the Fluo-8 containing cores (white) with different concentrations of pre-loaded $CaCl_2$ in levitated ACDC droplets. Scale bars denote 1mm. The white dotted ellipsoid in panel 1 indicates the perimeter of the whole ACDC droplet. Droplet was not locked by magnetic manipulation. Error bars indicate the standard error of mean of the relative intensity.

Relative fluorescence signal intensity v.s. evaporation. Droplet was not locked by magnetic manipulation.

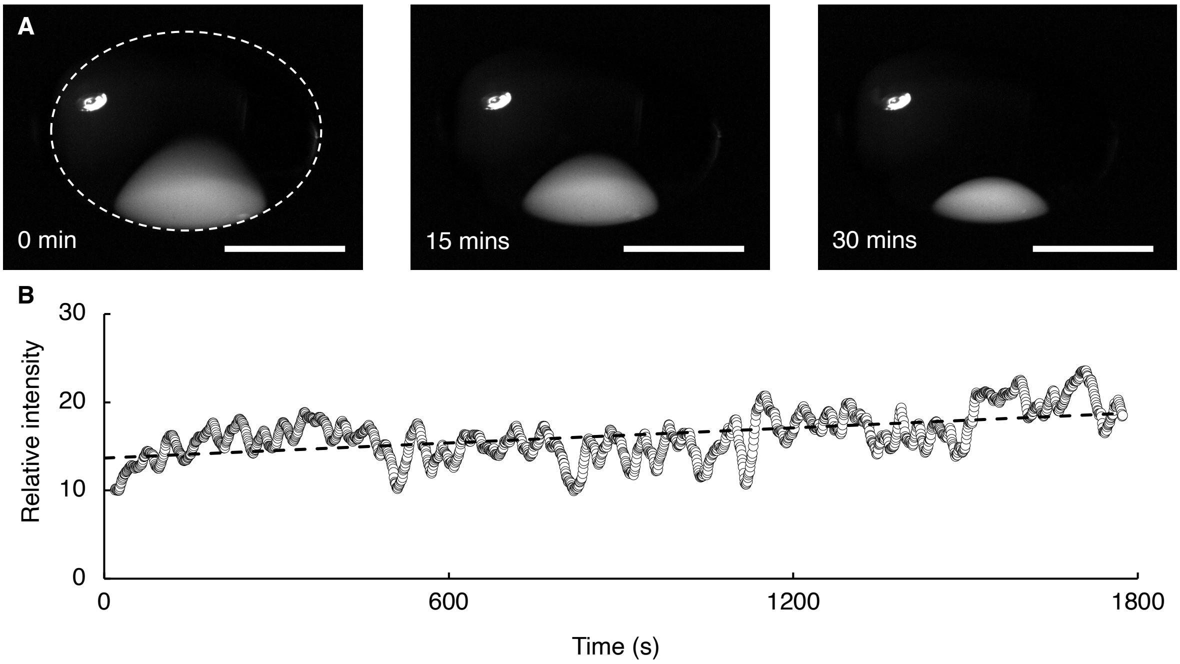

Fig S10. Evaporation effects on fluorescence intensity are minimal compared to Ca^{2+} transport over the course of 30 minutes. The volume loss and the relative intensity change due to concentrating of the fluo-8 within an aqueous core (fluorescent white). Core also contains 3.3 mM $CaCl_2$. Scale bars = 1 mm. The white dotted ellipsoid indicates the edge of the whole ACDC droplet. Droplet was not locked by magnetic manipulation.

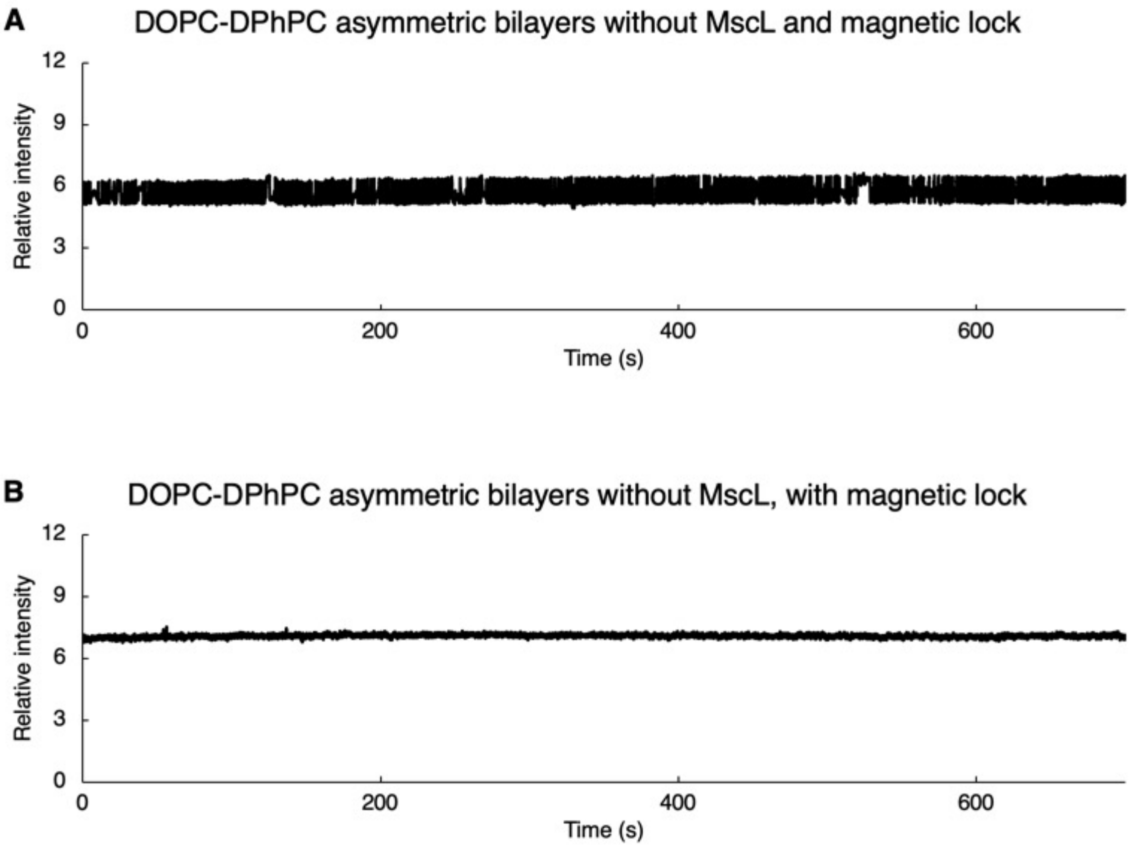

Fig S11. Control experiment of MscL gating by asymmetric DOPC-DPhPC bilayers. The droplet model is the same as shown in Fig 4B. **A.** Relative intensity change of the Fluo-8 containing core without MscL, without the droplet rotation locked by magnetic operation. **B.** Relative intensity change of the Fluo-8 containing core without MscL, with the droplet rotation locked by magnetic operation. These results indicated that the asymmetric bilayer leaflet did not spontaneously form pores in levitated ACDC droplets.

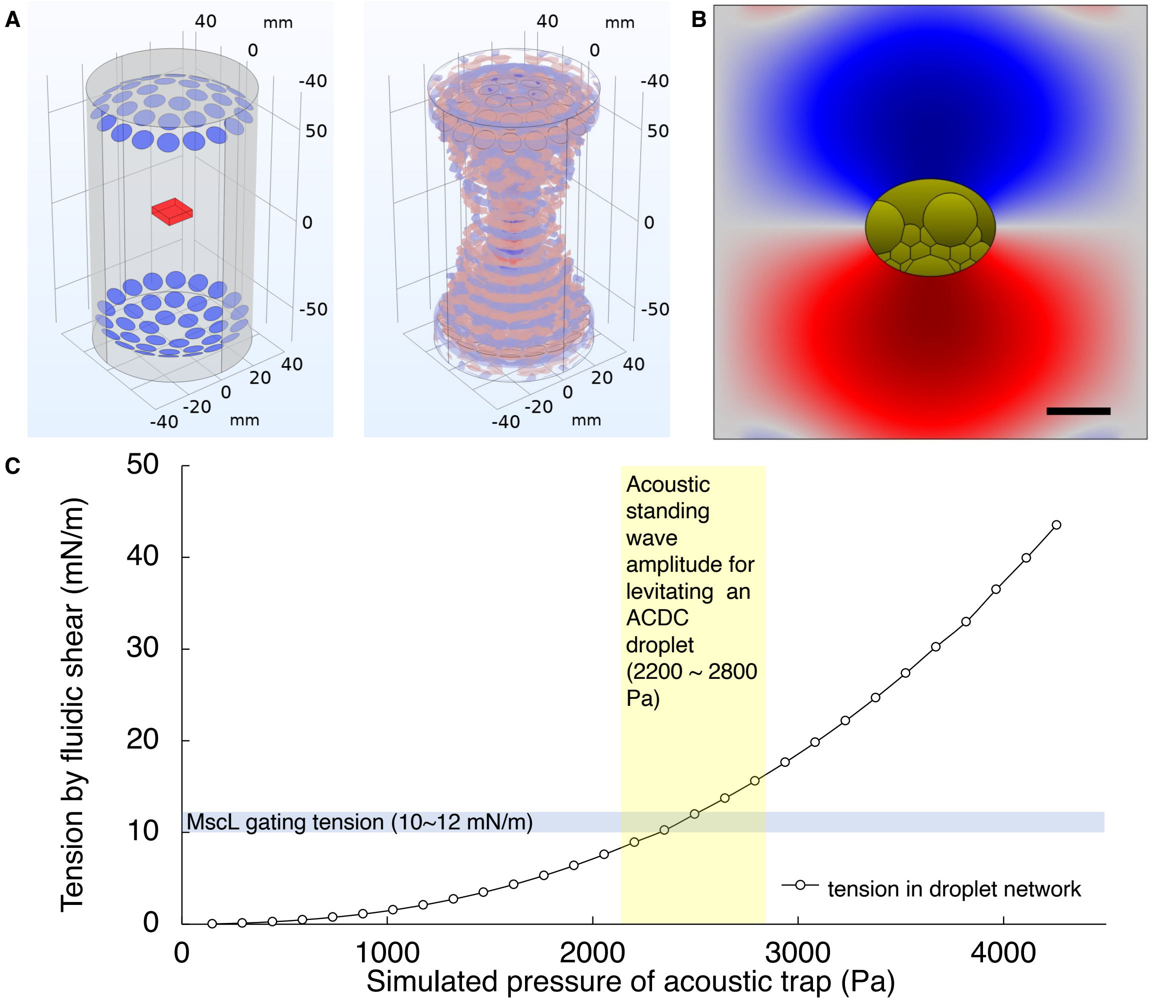

Fig S12. Simulated results of magnetic operation induced tension within the droplet network of levitated ACDC droplet. A. Multiphysics model of droplet acoustic levitation using pressure acoustics module and particle tracing module of COMSOL software. Left- the arrangement of pressure inputs (blue) is built upon our acoustic levitator transducers array; the red box indicates the region of simulated particle release to simulate droplet levitation in the pressure node of the acoustic standing wave as visualised on the right. This model is employed to calculate the pressure required to levitate ACDC droplets, which corresponds to the region of ~2200-2800 Pa. **B.** Multiphysics model used to model the acoustic streaming effect in levitated ACDC droplet, using the thermoviscous acoustics module, laminar flow module and particle tracing module of COMSOL software. The resultant fluid streamlines and profiles of convective flow are shown in Fig 5G. This model is employed to calculate the fluid flow shear rate and consequently the induced tension on all the aqueous droplet boundaries of ACDC droplet. Scale bar = 1mm. **C.** Simulated result of the induced tension on ACDC droplet network at different applied acoustic pressures (levitator power). The blue band indicates the membrane tension range for MscL gating reported in previous literature [2]. The yellow band highlights the acoustic pressure range to levitate ACDC droplets (simulated based on the acoustic levitator in this work). This simulation indicates the induced tension during acoustic and magnetic operation is in the range of 8~16 mN/m.

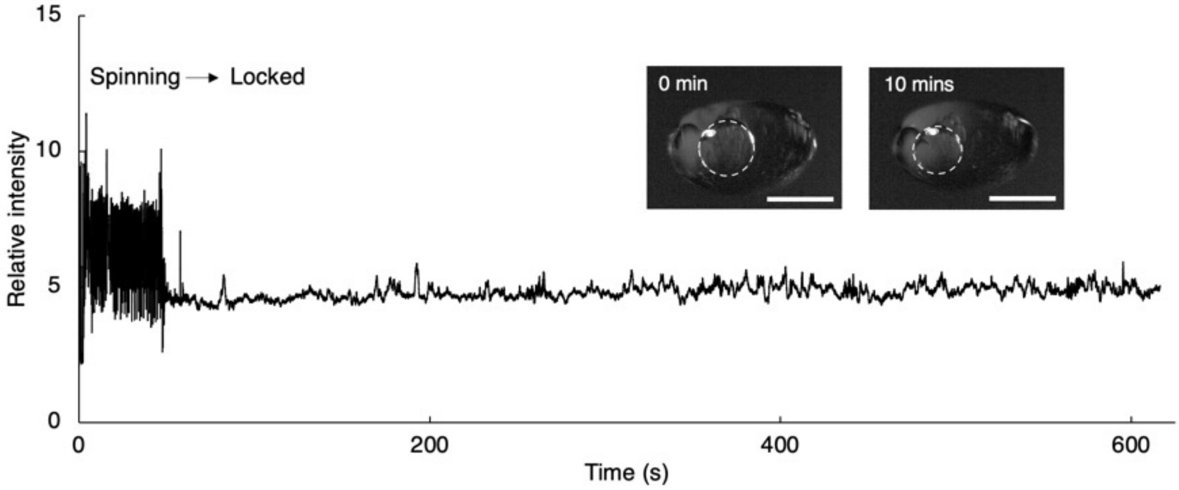

Fig S13. Relative intensity of a control core (white dotted circle) containing fluo-8 but no MscL, in a levitated ACDC droplet with symmetric DPhPC bilayer membranes. The droplet was locked by magnetic manipulation. The lipid bilayers remain impermeable, with no significant intensity increase was observed. This result is complementary to Fig 5. The experimental droplet contains equivalent buffer materials to the MscL protein preparation, including a final concentration of 4 μM DDM, confirming that measured activation in MscL containing droplets (Fig 4, 5 and 6) is protein mediated. Scale bars = 1mm.

Reference

1. Castell, O. K., Dijkman, P. M., Wiseman, D. N., & Goddard, A. D. Single molecule fluorescence for membrane proteins. *Methods* **147**, 221-228 (2018).
2. Berrier, C., Besnard, M., Ajouz, B., Coulombe, A., & Ghazi, A. Multiple mechanosensitive ion channels from Escherichia coli, activated at different thresholds of applied pressure. *The Journal of membrane biology* **151**, 175-187 (1996).
