# Supplementary material for: Building programmable multicompartment artificial cells incorporating remotely activated protein channels using microfluidics and acoustic levitation
Source: Nat Commun. 2022 Jul 15;13:4125. doi: 10.1038/s41467-022-31898-w (PMC9287423; doi:10.1038/s41467-022-31898-w)
Supplement: Supplementary file 3 — Description of Additional Supplementary Files [file 41467_2022_31898_MOESM3_ESM.pdf]

Description of Additional Supplementary Files

File Name: Supplementary Movie 1
Description: Acoustic levitation of microfluidically formed, multicompartment artificial cells.

File Name: Supplementary Movie 2
Description: Pneumatic, thermal magnetic, and light operation of levitated artificial cells.

File Name: Supplementary Movie 3
Description: Control of membrane protein gating in levitated artificial cells.
